# Synthesis of TiO_2_ with Hierarchical Porosity for the Photooxidation of Propene

**DOI:** 10.3390/molecules22122243

**Published:** 2017-12-16

**Authors:** Javier Fernández-Catalá, Laura Cano-Casanova, María Ángeles Lillo-Ródenas, Ángel Berenguer-Murcia, Diego Cazorla-Amorós

**Affiliations:** Inorganic Chemistry Department and Materials Institute, Alicante University, Ap. 99, E-03080 Alicante, Spain; j.fernandezcatala@ua.es (J.F.-C.); laura.cano@ua.es (L.C.-C.); mlillo@ua.es (M.Á.L.-R.); a.berenguer@ua.es (Á.B.-M.)

**Keywords:** TiO_2_, hierarchical porosity, photocatalytic activity, propene

## Abstract

The elimination of volatile organic compounds (VOCs) at low concentration is a subject of great interest because these compounds are very harmful for the environment and human health. In this work, we have developed a synthesis methodology of TiO_2_ that allows obtaining meso-macroporous materials with hierarchical porosity and with high thermal stability for their application as photocatalysts in the removal of VOCs, specifically propene. The materials synthesized in this work were characterized by Scanning electron microscope (SEM), Transmission electron microscopy (TEM), powder X-ray diffraction (XRD), Thermogravimetric Analysis (TG), and nitrogen adsorption. It is observed that the samples calcined at 250 °C and 500 °C present a high photoactivity for the photooxidation of propene, which is similar to the benchmark material P25 (commercial TiO_2_). Moreover, the textural properties are better than those for P25, indicating that the samples are interesting for the preparation of photocatalysts with different conformations, such as in the form of coatings and fillings in different size scales.

## 1. Introduction

At present, the removal of volatile organic compounds (VOCs) at low concentrations is still a hot topic, because these compounds are very harmful to the environment and human health. These pollutants cause carcinogenic effects, problems in the central nervous system and climate change, among several other adverse effects [1,2].

Among the different VOCs, the removal of propene is interesting because this contaminant is considered a highly reactive volatile organic compound (HRVOC) which is involved in the formation of ground-level and tropospheric ozone and, therefore, in photochemical smog [3,4]. This contaminant appears in vehicle emissions and in many industrial applications, such as petrochemical plants, cigarette smoke, and others [5,6]. There are several ways to eliminate propene, for example, incineration, adsorption, absorption, condensation, among others. However, heterogeneous photocatalytic oxidation (PCO) is a novel way to eliminate this pollutant because it can be performed at room temperature and at atmospheric pressures [7]. A wide variety of semiconductors has been studied as photocatalysts, such as ZnO [8], CdS [9] and, most noticeably, TiO_2_ [10,11,12].

Titania (TiO_2_) has received significant attention in the last decades for its unique properties, including photocatalytic activity, photo- and chemical stability, nontoxicity, and relatively low production cost [13,14,15]. Moreover, TiO_2_ is an important industrial product in many applications such as inorganic pigment, photocatalyst, sunscreen, or in energy storage and electrochromism [16,17,18,19,20]. From a structural point of view, titanium dioxide has three crystalline polymorphs: anatase, rutile, and brookite. Among the three crystalline phases, anatase shows a better photocatalytic behavior [21]. An interesting commercial TiO_2_ powder is P25 (EVONIK, Essen, Germany), which consists of 80% of the anatase phase and 20% of the rutile phase of titanium dioxide. This commercial titania nanoparticle is widely used as photocatalyst in photochemical reactions due to their very high photocatalytic activity [22] and is often used as benchmark material to assess the potential of different photocatalysts.

Many factors influence the photocatalytic properties of TiO_2_, such as the particle size, morphology, exposed lattice planes, and crystalline phase [23,24]. P25 has already been optimized in many of these factors. Nevertheless, this material presents comparatively low surface area and this factor may be important to improve the photooxidation of propene. For this reason, it is interesting to synthesize new TiO_2_ materials that improve on factors, such as the surface area and the capacity to protoxidize propene with respect to P25 [25,26,27]. Despite the fact that propene is a reasonably small molecule, if its mineralization is not complete, deposition of carbon on the catalyst surface cannot be ruled out and, thus, diffusional limitations may arise. Furthermore, the way TiO_2_ powders are usually produced (in the form of nanoparticles, as we have already reported [26]), the use of the catalyst in a bed configuration will likely result in compacting which will ultimately give rise to diffusional limitations. In order to ensure a proper mass transfer, a hierarchical catalyst architecture with linked macro-mesoporosity could be an interesting option.

In this work, we have synthesized several TiO_2_ samples with hierarchical porosity to obtain materials with large accessible surface area to improve the results obtained using P25 TiO_2_. These materials have been synthesized through modifications on the report by Zhu et al. [25] by incorporating urea in the sol synthesis in order to develop a material with pores large enough to warrant the absence of any diffusional limitations [28,29]. If this larger porosity can be enhanced by producing an interconnected network of meso- and macropores (which would give to a so-called hierarchical porosity) it would be of interest for the preparation of photocatalysts. It is of course also promising if such materials could be prepared with different conformations, such as in the form of coatings and fillings in different size scales for applications that need a robust continuous material and a good mass transfer for a good photooxidation performance [28]. Moreover, removal of the templates used during synthesis has been carried out by washing or calcination in order to check the effect of these treatments. The prepared materials were studied in photooxidation of propene at low concentrations at room temperatures and under UV irradiation, yielding promising results in terms of photocatalytic performance. 

## 2. Results and Discussion

### 2.1. Materials Characterization

#### 2.1.1. XRD Analysis

**Figure 1:**
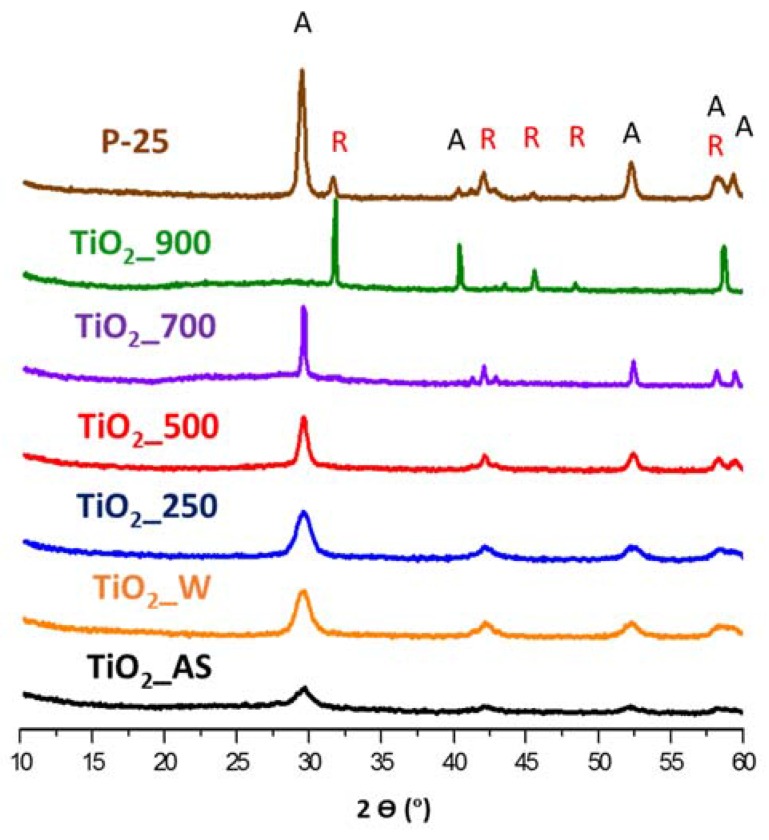
XRD patterns of TiO_2_ samples. Key: A = anatase; R = rutile.

The changes in crystal phase and crystallite size of all the samples were studied by ex situ powder X-ray diffraction (XRD), and the corresponding patterns of the samples calcined at different temperatures for 6 h are presented in Figure 1. The corresponding results regarding crystallite size and phase composition are listed in Table 1. In the XRD patterns it is observed that the as-synthesized TiO_2_ (without treatment) is composed of anatase phase with very low crystallinity. Upon increasing the calcination temperature in the 250–700 °C range the crystallinity of the samples increases (as evidenced from the sharper, narrower characteristic peaks of anatase TiO_2_) while maintaining the same anatase phase. As some authors have reported in the literature, the temperature up to which the anatase phase is stable is around 500 °C [30]. Treating the sample at higher temperatures results in the formation of the more stable rutile phase. In our case, the anatase-to-rutile transformation occurs in a significantly higher temperature range, between 700 °C and 900 °C, which indicates a very high thermal stability for the TiO_2_ samples prepared in this work. Given our modification of the reported protocol (see Sample Characterization (Section 3.3.) for full details), it appears that urea favors the thermal stabilization of the anatase phase in agreement with [29].

With respect to the crystallite size of the samples shown in Table 1, the washing and low-temperature calcination treatments did not affect the crystallite size, being 7 nm in three cases (as-synthesized, washed, and calcined at 250 °C). Nevertheless, increasing the calcination temperature results in the sintering and growth of larger crystals. Rietveld analyses of the XRD patterns may yield additional useful information, such as the microstrains in the particles as a result of the calcination process as other authors have reported [31], but the resolution of our spectra was not suitable to perform a reliable analysis.

#### 2.1.2. Thermogravimetric Analysis

**Figure 2:**
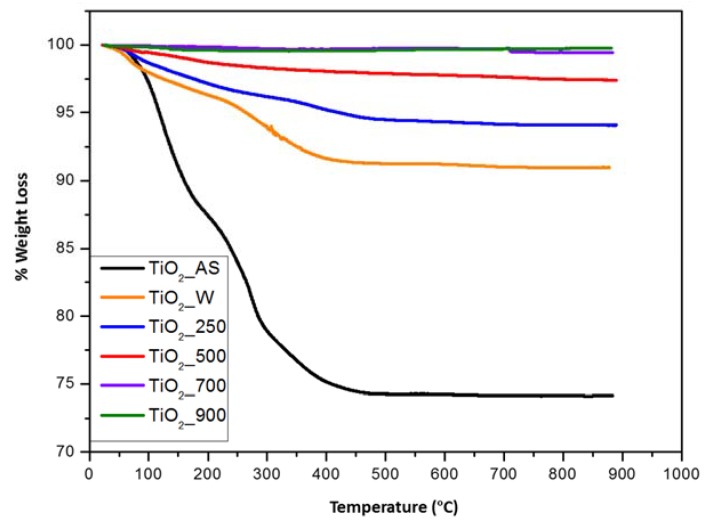
TG curves of the TiO_2_ samples prepared in this study.

In order to quantify the amount of organic matter present in the prepared samples, thermogravimetric analysis (TG) was done in all samples. The results of the performed TG experiments are showed in Figure 2. The as-synthesized TiO_2_ samples had a weight loss of about 25.8%, but samples TiO_2__W and TiO_2__250 already presented weight losses of around 9.0% and 6.0%, respectively, which are indicative of the removal of a substantial part of the organic matter used during the synthesis. The complete removal of the template requires higher temperatures and this is necessary since they may block the porosity, affecting the (photo) catalytic performance. At calcination temperatures of 500 °C, 700 °C, and 900 °C these values were 2.5%, 0.58%, and 0.25%. The weight loss detected for the sample calcined at 500 °C could be due to the removal of traces of organic matter and weakly-adsorbed water at lower temperatures [32]. In order to corroborate this observation, FTIR experiments were conducted for the prepared TiO_2_ samples (vide infra).

#### 2.1.3. FT-IR Spectroscopy

**Figure 3:**
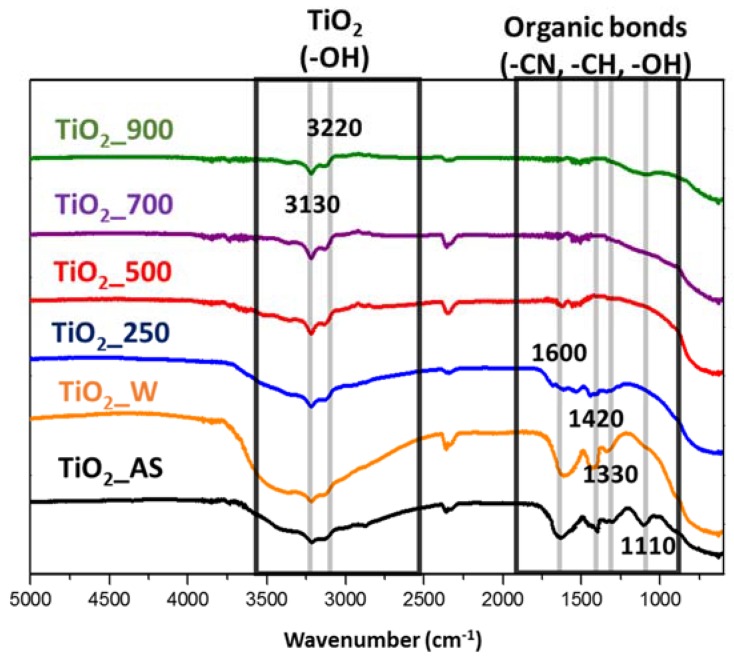
FTIR spectra of the TiO_2_ samples prepared in this study.

The analysis of the surface chemistry and the removal of reagents/templates (organic matter) after heat treatment were analyzed by FTIR. The results are presented in Figure 3. The broad band at about 3000–3500 cm^−1^ and 1600 is attributed to the OH stretching of physisorbed water on the TiO_2_ surfaces and hydrogen-bonded hydroxyl groups [33]. The as-synthesized sample and the washed sample show this broad band. After calcination at 250 °C the bands at 3000–3500 and 1600 cm^−1^ decreased in intensity, indicating the loss of physisorbed water on the TiO_2_ surface. Moreover, when the calcination temperature is further increased this adsorbed water is eliminated and the bands appearing at 3000–3500 and 1600 cm^−1^ disappear. The remaining bands appearing at 3130 and 3220 cm^−1^ are indicative of the presence of OH groups on the surface of the TiO_2_ [34]. This result agrees with the TG analyses in which the samples washed and calcined at 250 °C presented noticeable weight losses up to 500 °C, probably due to the removal of water and organic residues at lower temperature and adsorbed water at higher temperatures. In this respect, as the FTIR results show, increasing the calcination temperature not only removes the template molecules along with any remaining organic reagents used (lower wavenumber region), but also removes the physisorbed water (both weakly and strongly adsorbed water [32], higher wavenumber region) which might be detrimental towards photocatalytic applications.

The bands at 1110 cm^−1^ and 1420 cm^−1^ in the as-synthesized sample are assigned to the stretching vibration of C-N and the presence of the deformation mode of ammonium ions formed by the decomposition of excess urea [29], respectively. Moreover, this sample also presents bands at 1420, 1330, and 1110 cm^−1^ corresponding to C–H [11] vibrations of surfactants and complexing agents. In the sample washed with ethanol the band at 1110 cm^−1^ is not present because this step removed a significant amount of organic reagents but, as indicated in the TG experiments (Figure 2), not all organic matter was removed in the washing step. When the sample was calcined at low temperature (TiO_2__250) the bands corresponding to templates (organic matter) decreased. For this reason, and following up on the FTIR and TG results, it is necessary to calcine at 500 °C or higher temperatures in order to eliminate all reagents. Washing or calcination at lower temperatures may result in porosity blocking and/or adverse effects in the surface chemistry of the final material. These results are consistent with TG experiments, because the as-synthesized TiO_2_ has the higher loss of organic matter and presents the relevant characteristic bands for the corresponding compounds, as discussed above.

#### 2.1.4. UV–VIS Spectroscopy

**Figure 4:**
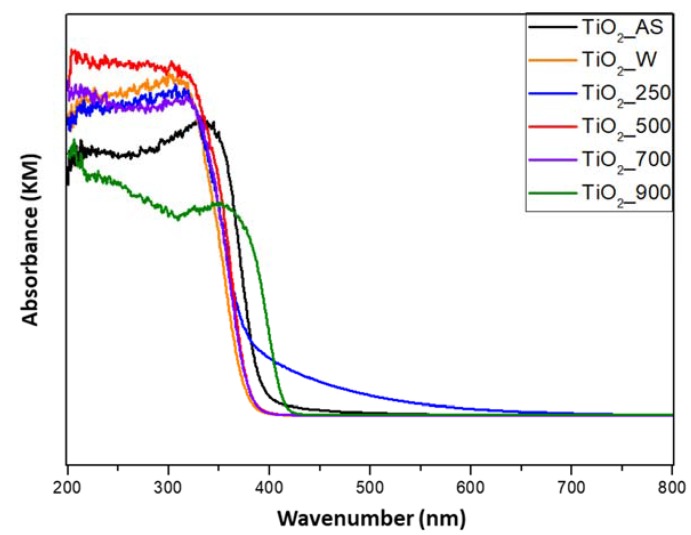
UV–VIS absorption spectra of the TiO_2_ prepared samples.

The absorption of the TiO_2_ materials prepared in this work was evaluated by UV–VIS spectroscopy. The results are presented in Figure 4 and in Table 2. The samples with the crystal phase anatase present a similar absorption edge and band gap, which is typical for anatase. However, the as-synthesized TiO_2__sample shows a slightly smaller band gap. This might be attributed to the very low crystallinity of the anatase phase and the presence of the templating agents used during the synthesis as shown in the XRD analysis, TG experiments, and FTIR spectra experiments. Sample TiO_2__900 (the pure rutile phase from our XRD results) present a different absorption edge and band gap (414 λ and 2.99 eV, respectively) with respect to the other samples, which is characteristic for the rutile phase.

#### 2.1.5. Porosity Characterization

**Figure 5:**
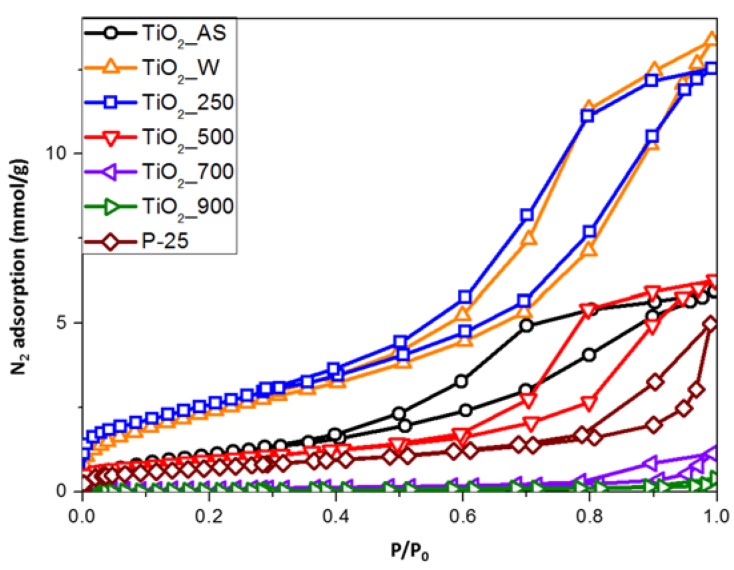
Isotherms of N_2_ at 77 K for the different TiO_2_ samples prepared in this study. The adsorption isotherm of the benchmark material P25 is also shown for comparison purposes.

Nitrogen adsorption-desorption measurements were performed to determine textural properties of the TiO_2_ samples. As revealed in Figure 5, the isotherms are of type IV with a hysteresis which is typical of mesoporous materials. The N_2_ uptake and the size of the hysteresis loop is dependent on the treatment applied. In this sense, samples TiO_2__W and TiO_2__250 exhibit the highest uptakes, but present a small uptake at low relative pressures, which is indicative of a small amount of microporosity. Thus, both washing and calcination at a low temperature are able to activate the samples almost to a full extent, even if some organic moieties are still present (vide supra). Samples TiO_2__700 and TiO_2__900 show a significant interparticle sintering and collapse of the porous structure, as evidenced from their small surface areas. However, sample TiO_2__500 presents an isotherm close to that of sample TiO_2__AS, but, as shown in the TG experiments (Figure 2), the presence of leftover template/chemicals is very low.

Concerning the textural properties shown in Table 3, as the calcination temperature is increased, the BET surface area decreases from ~200 m^2^/g (samples TiO_2__W and TiO_2__250) to 4 m^2^/g (sample TiO_2__900). It, thus, appears that while sufficiently high calcination temperatures are needed in order to completely remove the organic matter, the very small TiO_2_ crystallites have a strong tendency to sinterize (even at reletively low temperatures) given their high surface energy and the TiO_2_ structure collapses at temperatures ≥700 °C. This, however does not compromise the thermal stability of the anatase phase, which might be due to the synthesized samples being constituted by very small crystallites (see below, TEM analysis) with a large population of defects (probably as a consequence of the urea synthesis method employed) which block the anatase-to-rutile transition [30]. A similar trend is also observed for the total pore volume and the total micropore volume calculated applying the DR equation to the N_2_ adsorption data, which indicates the collapse of the structure as the sample is heated to higher temperatures. If we compare the prepared samples with the benchmark solid P25, as included in Table 3, it is shown how the samples calcined up to 500 °C possess higher surface area and pore volume.

#### 2.1.6. Electron Microscopy Characterization

**Figure 6:**
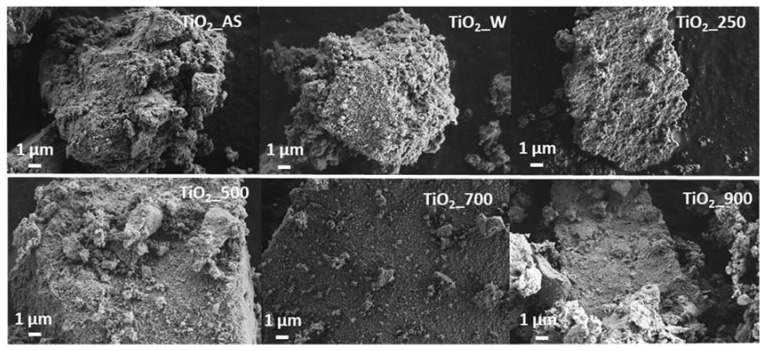
SEM of the different TiO_2_ samples prepared in this study. Scale bar: 1 μm.

**Figure 7:**
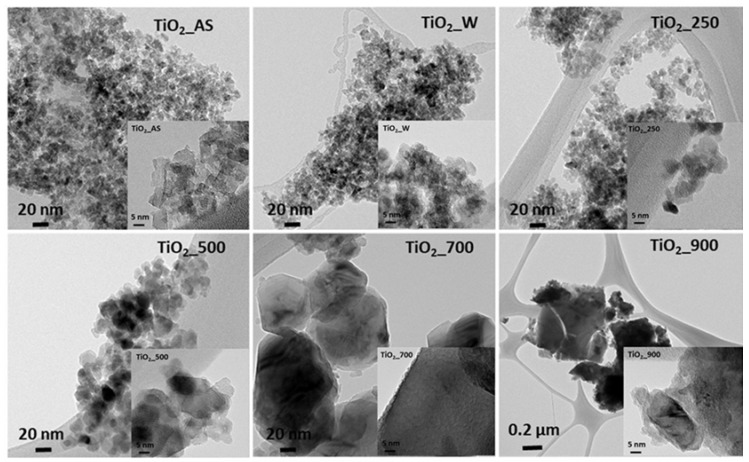
TEM of the different TiO_2_ samples prepared in this study. Insets: higher magnification images for all samples. Scale bar for all images except TiO_2__900: 20 nm; Scale bar for sample TiO_2__900: 0.2 μm. Scale bars for insets: 5 nm.

The morphology of the TiO_2_ samples prepared in this study is shown in Figure 6 (SEM images) and Figure 7 (TEM images). As shown in Figure 6, all samples present a heterogeneous morphology. The rougher surface observed for the as-synthesized sample, as well as those for samples calcined at temperatures below 500 °C, seems to become smoother upon calcination at higher temperatures, which might be indicative of the sintering of the crystallites and the collapse of the structure. Upon observation under TEM the samples consist of the aggregation of nanoparticles synthesized during the sol-gel process. Samples TiO_2__AS, TiO_2__W, and TiO_2__250 have similar mean particle sizes, which agrees very well with the crystallite sizes determined from XRD analyses. At higher calcination temperatures, the mean particle size noticeably increases (Figure 7 and Table 4) in agreement with the literature [26]. The insets in Figure 7 show higher magnification pictures taken for the samples prepared in this study, in which a small surface amorphous layer is visible. This layer becomes less noticeable as the calcination temperature is increased. Given our observations, this amorphous layer might be similar to that previously reported [35] for inorganic-organic core-shell TiO_2_ materials prepared by the combustion sol-gel method. It must be emphasized that, from the SEM and N_2_ adsorption results, the samples prepared in this work present a hierarchical structure, as it can be evidenced by the large difference between the total pore volumes with respect to the values reported in the literature for similar solids [26]. It must be noted that, differently from our previous studies, the reported synthetic strategy did not rely on the precipitation of TiO_2_ nanoparticles based on a sol-gel route, but on the transformation of a TiO_2_ hydrogel into a TiO_2_ hierarchical monolithic structure as a continuation of our previous efforts in which we have prepared continuous SiO_2_ monoliths and coatings following a very similar approach [28] for applications needing a robust continuous material. In this respect, the materials prepared in this study are comprised of nanoparticles which have formed from a gel and which are organized in two different levels: one around a nonionic surfactant, and the other arising from the decomposition of urea during the second step of the hydrothermal treatment. Comparing the particle size in TEM with the crystallite size obtained by XRD (Table 1), it is observed that at high temperatures there are significant discrepancies between the observed crystallite size and that determined by XRD. This is ascribed to the fact that the particles observed in TEM are not TiO_2_ single crystals, but aggregates comprised of several TiO_2_ smaller crystals which preserve their domains upon sintering. 

#### 2.1.7. In Situ XRD Analyses

**Figure 8:**
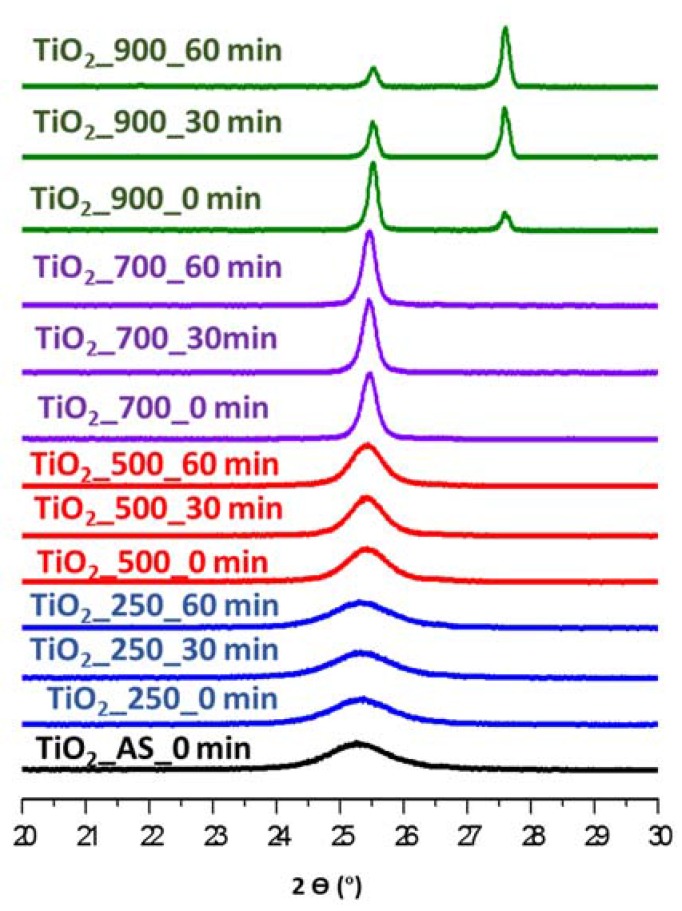
In situ XRD patterns at different temperatures of TiO_2_ samples.

In order to gain a better insight on the thermal stability of the prepared TiO_2_ samples, in situ XRD measurements were carried out. Sample TiO_2__AS (as-synthesized) was mounted in the holder and a first scan was obtained at room temperature. The sample was then treated as described in the Experimental section, collecting XRD spectra at the indicated temperatures and times. The results corresponding to the regions where the two characteristic peaks for anatase and rutile appear are presented in Figure 8. Table 5 shows the phase composition of the samples after each respective treatment and the corresponding crystallite size. In Figure 8 it is observed that the only phase for the starting sample TiO_2__AS is pure anatase, whose crystallinity increases with increasing temperature. The anatase to rutile transition takes place between 700 °C and 900 °C, which is a high temperature compared with the literature [30,36]. Moreover, when heating at temperatures ≥500 °C the size of the crystallites grows over time, as it can be seen comparing the results from the spectra obtained at 0 and 60 min of dwelling time. In this respect, the calcination time is an important tool to control the final crystallite size, as shown in Table 5. 

With respect to the phase composition, up to 700 °C all samples with different dwelling time present only the anatase phase. However, upon reaching 900 °C the phase composition of the sample changes over time. Initially (no dwelling time) sample TiO_2__900 is composed of 75% anatase and 25% rutile (similar to P25, that presents 80% anatase and 20% rutile). Moreover, if one compares this XRD in-situ at 900 °C with the XRD at 900 °C shown in Figure 1 it is clear that the same sample calcined at 900 °C for 6 h has completely undergone the anatase-to-rutile transition, indicating once again how calcination time is an important parameter for the preparation of the samples in terms of both phase composition and crystal size.

### 2.2. Photocatalytic Activity

**Figure 9:**
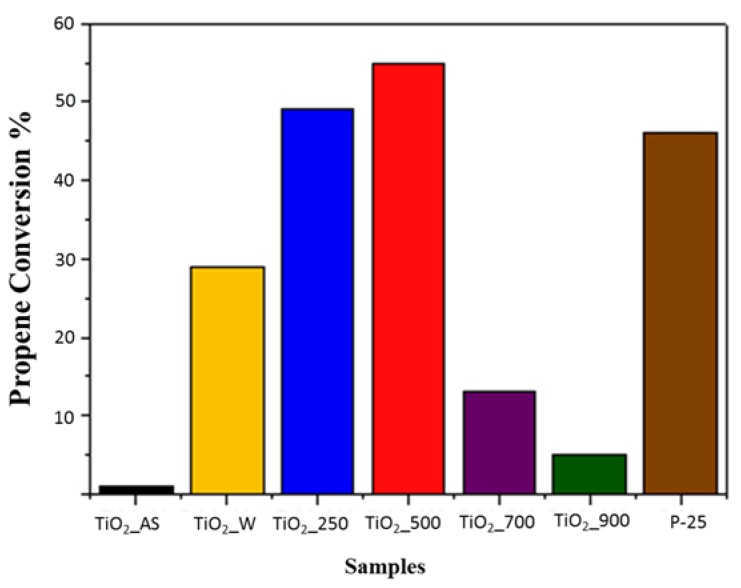
Conversion of propene for TiO_2_ samples and P25.

The photocatalytic activity of TiO_2_ materials prepared in this work was evaluated by studying the mineralization of propene in the gas phase (100 ppmv (parts per million by volume) in air) to CO_2_ at room temperature, following the reaction shown in Equation (1) [37]. Given that the photocatalyst used is no different from both a chemical and structural point of view, the mechanism responsible for the photooxidation process is the one already reported in literature [15,38]. Additionally, P25 was also used as a reference material. The results are summarized in Figure 9.

2C_3_H_6_ + 9O_2_ ↔ 6CO_2_ + 6H_2_O (1)

The as-synthesized TiO_2_ sample did not show photooxidation of propene because the TiO_2_ active sites were blocked to reactants due to the presence of urea and the templating agent and/or the fact that the TiO_2_ structure was not consolidated. When the sample was washed with EtOH under reflux the photoactivity increases noticeably, but the conversion is still below the performance of the benchmark P25 catalyst. This might be due to the fact that the washing does not remove all the organic matter (as evidenced by TG analysis) and does not increase the crystallinity (as shown by XRD). The porosity of the sample is more accesible (BET surface are over 200 m^2^/g) but the structure is still not sufficiently consolidated and the remaining organic matter may be affecting the catalytic activity. The sample calcined at lower temperature (250 °C) presents a good conversion of propene (around 50%) which surpasses the conversion of propene observed for sample P25. However, this sample has some residues from the template and a similar crystallinity to the washed sample. The main difference between these two samples is adscribed to the presence of water in the washed sample, which is detrimental in the photocatalytic activity of the resulting TiO_2_. The sample calcined at 500 °C presents the best photocatalytic activity reported in this study. This sample present lower porosity compared to the samples washed or calcined at 250 °C, and a noticeable sinterization of particles with respect to sample TiO_2__250 (from the TEM analysis). However, sample TiO_2__500 presents an improved crystallinity over these two samples and the absence of any organic impurities and adsorbed water (FTIR). These factors can improve the photocatalytic activity. For the samples calcined at 700 °C and 900 °C, the propene conversion decreases sharply because of the sinterization of the particles and the collapse of the porous structure. 

Considering the results obtained in this study in terms of crystal size, porous texture, and surface chemistry, it would appear that a compromise solution is mandatory in order to come up with the sample displaying the best performance. The most promising outlook seems to be the following: (1) large surface areas are beneficial for an enhanced access of reagents to the active sites of the photoactive material; (2) water adsorbed on the surface of the TiO_2_ impoverishes the photocatalytic activity since it may enhance the recombination rate of electron-hole pairs [39]; (3) high crystallinities translate into improved photocatalytic activity. In this respect, the samples which show the highest surface area values do not present adequate surface chemistry (i.e., large bands in the 3000–3300 cm^−1^ region evidence a large amount of adsorbed water), have some organic matter or do not have a sufficiently crystalline structure. Thus, the obtained results are below those expected for a high surface area TiO_2_ sample. On the other hand, the samples calcined at higher temperatures present a significantly more crystalline structure, but their surface area is greatly diminished, which undermines their photocatalytic performance to a very large extent. In this respect, the sample calcined at 500 °C presents the best interplay because it still possesses a BET surface area of around 85 m^2^/g together with a structure showing sufficient crystallinity and appropriate surface chemistry without organic matter, as shown by XRD, TG, and FTIR. This, in turn, results in a material which can be prepared by a simple, reproducible, and cost-effective sol-gel method which displays a remarkable photocatalytic activity.

## 3. Materials and Methods

### 3.1. Materials

Titanium (IV) butoxide (TTB, 97%, Sigma-Aldrich, St. Louis, MO, USA), glacial acetic acid (HAc, 99%, Sigma-Aldrich), Pluronic F-127 (F-127, Sigma-Aldrich), absolute ethanol (EtOH, 99.8%, Fisher Scientific, Hampton, NH, USA), formamide (FA, 99.5%, Sigma-Aldrich), urea (99%, Merck, Kenilworth, NJ, USA), and deionized water were used in the present work. All reactants were used as received, without further purification.

### 3.2. Sample Preparation

The TiO_2_ samples were prepared adapting a previously reported synthesis performed by Zhu et al. [25]. The main difference was the incorporation of urea in order to induce the desired formation of mesoporosity, through the gradual decomposition of urea giving rise to a controlled and homogeneous pH variation as reported by our research group which has successfully given rise to robust hierarchical silica monoliths [28].

As an illustrative example, the synthesis of TiO_2_ samples was performed as follows: 5 g of the titanium precursor (titanium tetrabutoxide, TTB) were weighed and dissolved in 7.9 g of EtOH. This solution (“solution A”) was stirred vigorously for 10 min. Then, in this order 1.6 g of HAc, 0.3 g of F-127, 1.6 g of deionized water, 7.9 g EtOH, 0.4 g of FA, and 0.4 g of urea were weighed and added in a separate vessel. The mixture was stirred for 10 min (“solution B”). Solution B was added dropwise on “solution A” under vigorous stirring. The resulting solution was rapidly transferred to an autoclave and heated at 60 °C for 24 h to promote gel formation and the temperature was later increased to 120 °C with a dwelling time of 24 h to promote the decomposition of urea. The samples obtained were washed or calcined at different temperatures. The nomenclature of the samples used in this work were TiO_2__AS for sample without treatment (as-synthesized), TiO_2__W for sample washed with ethanol for 9 h under reflux conditions and TiO_2__250, TiO_2__500, TiO_2__700, and TiO_2__900 for samples calcined at 250 °C, 500 °C, 700 °C, and 900 °C, respectively, for 6 h with a heating ramp rate of 1 °C/min.

### 3.3. Sample Characterization

Several techniques were employed for the characterization of the samples. The crystal phase composition and crystallinity of TiO_2_ were determined by X-ray diffraction (XRD) analysis using a SEIFERT 2002 equipment. Cu Kα (1.54 Å) radiation was used. The scanning velocity was 2°/min, and diffraction patterns were recorded in the angular 2θ range of 6–80°. The crystallite size was estimated by applying the Scherrer equation (Equation (2)) [40] using the full width at half-maximum (FWHM) data of the major diffraction peak and a K factor of 0.93:

(2) $$B=\frac{K\lambda }{\beta \cos\theta }$$

where B is the crystalline size (nm); K is the dimensionless shape factor whose value is 0.93; λ is the wavelength of the radiation source used, which is 1.54056 Å for Cu Kα radiation; β is the full width at half maximum intensity (FWHM) (radians), and θ is the Bragg angle at the position of the peak maximum. We used an internal standard (Silicium carbide, SiC) to correct the effect of both the equipment and the analysis temperature. 

The surface chemistry of the TiO_2_ materials was analyzed by Fourier transform infrared (FTIR) spectroscopy (FTIR JASCO 4100, Pfungstadt, Germany) in transmission mode from 400 cm^−1^ to 4000 cm^−1^.

The optical absorption properties were studied by UV-VIS/DR spectroscopy (Jasco V-670, Pfungstadt, Germany, with an integrating sphere accessory and powder sample holder). BaSO_4_ was used as the reference standard and the reflectance signal was calibrated with a Spectralon standard (Labsphere SRS-99-010, North Sutton, NH, USA, 99% reflectance). The absorption edge wavelength was estimated from the intercept at zero absorbance of the high slope portion of each individual spectrum in the range 200–800 nm (absorbance method). Then, the band gap can be calculated [41] as:

(3) $$\mathrm{Eg}=\frac{1239.8}{\lambda }$$

where Eg is the band gap energy (eV) and λ is the edge wavelength (nm).

The organic residue contained in the TiO_2_ samples (due to any template leftovers) was determined by thermogravimetric analysis using a thermobalance (SDT 2960 instrument, TA, New Castle, DE, USA,). In these analyses, the sample was heated up at 900 °C in air (heating rate of 5 °C min^−1^).

Textural properties were determined by applying the Brunauer-Emmett-Teller (BET) equation, and the Dubinin-Raduskevich equation to the N_2_ adsorption data obtained at −196 °C using an Autosorb-6B apparatus from Quantachrome (Boynton Beach, FL, USA) [42], obtaining the BET surface area (S_BET_) and total micropore volume (*V*_N_2__), respectively. The as-synthesized and washed TiO_2_ samples were degassed at 110 °C for 11 h and the calcined TiO_2_ samples were degassed at 250 °C for 4 h prior to adsorption experiments. Pore volume and average pore size were determined by nitrogen adsorption volume at a relative pressure of 0.99. Mesopore size distributions for all the samples were obtained applying the Barrett-Joynet-Halenda (BJH) formula to the N_2_ desorption branch data from the adsorption isotherms at −196 °C, using the software provided by Quantachrome. 

The morphology of the samples was analyzed by transmission electron microscopy (TEM, JEOL JEM 2010, Peabody, MA, USA) and field emission scanning electron microscope (FESEM, ZEISS Merlin VP Compact, Jena, Germany). Average nanoparticle size was carried out by counting 100 particles (except for samples TiO_2__700, TiO_2__900, for which 50 and 30 particles were counted, respectively) for each sample. The evolution of crystal phase composition and crystallinity of TiO_2_ was determined by X-ray diffraction (XRD) in situ, change the temperature using a Bruker D8-Advance equipment. Cu Kα (0.15418 nm) radiation was used. The scanning velocity was 2°/min, and diffraction patterns were recorded in the angular 2θ range of 6–60°. SiC was used to correct the effect of temperature. The ramp used in this experiment was the following, the sample was heated to 25 at 900 °C (heating rate of 5 °C min^−1^) measuring at 25, 250, 500, 700, and 900 °C for dwelling times of 0, 30, and 60 min in these temperatures. The crystallite size was estimated by applying the Scherrer Equation (2). The content of anatase was also calculated by applying the Spurr-Myers equation, as shown in Equation (4) [43]:

(4) $$W_{A}=\frac{1}{1+1.26I_{R}/I_{A}}\;W_{R}(\%)+W_{A}(\%)=100$$

where W_A_ is the weight fraction of anatase in the mixture; W_R_ is the weight fraction of rutile in the mixture; I_R_ is the intensity of the diffraction peak of rutile; and I_A_ is the intensity of the diffraction peak of anatase.

### 3.4. Catalytic Test

The photocatalytic performance of the different materials was studied using an experimental system designed in our laboratory. The system consists of a vertical quartz reactor where the photocatalyst bed is placed on a quartz wool bed. The reactor is 50 mm in height, its diameter is 20 mm, and the quartz wool support height is around 10 mm. A UV lamp is placed parallel to the quartz reactor, at a distance around 1 cm. The UV lamp radiation peak appears at 365 nm. The commercial reference of this lamp is a TL 8W/05 FAM (Philips, Amsterdam, The Netherlands, 1W). Finally, the coupled quartz reactor lamp is surrounded by a cylinder covered with aluminum foil. A scheme of this system is detailed elsewhere [35].

The weight of photocatalyst used in these experiments was 0.11 g for all the samples. The photocatalysts were used for the oxidation of propene at 100 ppmv in air at room temperature, 25 °C. The calibrated gas cylinder was supplied by Carburos Metálicos, S.A. (Cornellà de Llobregat Barcelona, Spain) The flow rate of the propene-containing stream was 30 (STP) mL/min in all experiments.

The propene-containing stream was passed through the photocatalyst bed and, afterwards it was injected in a GC chromatograph (Agilent 6890N, Santa Clara, CA, USA) working with a CTR-I column (Alltech, Sabadell, Barcelona, Spain) at 30 °C. GC chromatograph permits to follow the evolution of the concentration of propene in the outlet gas. Propene conversion was calculated using the flowing expression:

(5) $$\mathrm{Propene}\;\mathrm{conversion}\left(\%\right)=\frac{C_{{\mathrm{initial}}_{C_{3}H_{6}}}-C_{{\mathrm{stationary}}_{C_{3}H_{6}}}}{C_{{\mathrm{initial}}_{C_{3}H_{6}}}}\times 100$$

where C_initialC_3_H_6__ is the initial propene concentration, 100 ppmv and C_stationaryC_3_H_6__ is the propene concentration at steady state conditions in the photocatalyst bed outlet gas when the UV light is switched on.

Additionally, blank tests were performed under the same experimental conditions as the catalytic tests, but in the absence of the TiO_2_ photocatalysts, and no catalytic activity was detected.

## 4. Conclusions

In this study, a strategy of synthesis of TiO_2_ of hierarchical porosity has been developed through modification of the synthesis of Zhu et al. The protocol allows obtaining meso-macroporous materials which may be applied in the photocatalytic elimination of propene. Samples TiO_2__250 and TiO_2__500 prepared in this study have a high photocatalytic activity for propene photooxidation. Their performance is similar, and even slightly higher, to P25 (commercial TiO_2_), indicating that the TiO_2__250 and TiO_2__500 samples are interesting for the preparation of photocatalysts with different conformations, such as in the form of coatings and fillings in different size scales furthermore show a good photooxidation performance at low concentrations. Interestingly, the nanostructure generated by this methodology delays the conversion of anatase to rutile to temperatures higher than 700 °C. Probably, the mesoporosity induced by the presence of urea generates a large concentration of defects that slows down the phase transition rate. 

## Figures and Tables

**Table 1:** Crystallite size and phase composition of the TiO_2_ samples prepared in this study (see Sample Characterization (Section 3.3) for full details).

Samples	Crystal Phase Composition	Crystallite Size (nm)
TiO_2__AS	100% Anatase	6
TiO_2__W	100% Anatase	7
TiO_2__250	100% Anatase	7
TiO_2__500	100% Anatase	13
TiO_2__700	100% Anatase	26
TiO_2__900	100% Rutile	48

**Table 2:** Absorption edge wavelengths (nm), band gap (Eg) and crystal phase composition values of the TiO_2_ samples prepared in this study. The data for benchmark material P25 are shown for comparison purposes. Key: A = anatase; R = rutile.

Samples	λ (nm)	Eg (Ev)	Crystal Phase Composition
TiO_2__AS	391	3.17	100% A
TiO_2__W	377	3.29	100% A
TiO_2__250	390	3.17	100% A
TiO_2__500	383	3.23	100% A
TiO_2__700	381	3.25	100% A
TiO_2__900	414	2.99	100% R
P25	409	3.03	80% A + 20% R

**Table 3:** Textural properties of the TiO_2_ samples prepared in this study derived from the analysis of the N_2_ adsorption isotherms. The data for benchmark material P25 are shown for comparison purposes.

Samples	S_BET_ (m^2^/g)	V_total,0.95_ (cm^3^/g)	V_N2DR_ (cm^3^/g)	Mean Pore Size (nm)
TiO_2__AS	96	0.20	0.04	6.2
TiO_2__W	201	0.43	0.07	8.8
TiO_2__250	212	0.42	0.08	6.2
TiO_2__500	85	0.20	0.03	8.7
TiO_2__700	7	0.02	0.00	-
TiO_2__900	4	0.01	0.00	-
P25	55	0.18	0.02	7.6

**Table 4:** Crystalline size with TEM and XRD of TiO_2_ samples. The mean crystallite size from TEM analysis was performed by counting 100 separate particles (unless stated otherwise) and averaging the results.

Samples	Crystallite Size (nm) “TEM”	Crystallite Size (nm) “XRD”
TiO_2__AS	6.4 ± 1.0	6
TiO_2__W	7.1 ± 0.9	7
TiO_2__250	7.2 ± 0.9	7
TiO_2__500	12.9 ± 1.5	13
TiO_2__700	80 ± 20 (50 part.)	26
TiO_2__900	270 ± 90 (30 part.)	48

**Table 5:** Phase composition and crystallite size of TiO_2_ samples obtained in XRD in situ at different temperatures and dwelling times. A = anatase; R = rutile.

Samples	Phase Composition	Anatase Crystallite Size (nm)
TiO_2__AS_0 min	A (100%)	7
TiO_2__250_0 min	A (100%)	7
TiO_2__250_30 min	A (100%)	7
TiO_2__250_60 min	A (100%)	7
TiO_2__500_0 min	A (100%)	11
TiO_2__500_30 min	A (100%)	13
TiO_2__500_60 min	A (100%)	14
TiO_2__700_0 min	A (100%)	31
TiO_2__700_30 min	A (100%)	33
TiO_2__700_60 min	A (100%)	34
TiO_2__900_0 min	A (75%) + R (25%)	48
TiO_2__900_30 min	A (35%) + R (65%)	49
TiO_2__900_60 min	A (3%) + R (97%)	51
